# Optimal Policy of Multiplayer Poker via Actor-Critic Reinforcement Learning

**DOI:** 10.3390/e24060774

**Published:** 2022-05-30

**Authors:** Daming Shi, Xudong Guo, Yi Liu, Wenhui Fan

**Affiliations:** Department of Automation, Tsinghua University, Beijing 100084, China; gxd20@mails.tsinghua.edu.cn (X.G.); yiliu@mail.tsinghua.edu.cn (Y.L.); fanwenhui@tsinghua.edu.cn (W.F.)

**Keywords:** multi-agent, reinforcement learning, Actor-Critic, poker, multi-player, optimal policy

## Abstract

Poker has been considered a challenging problem in both artificial intelligence and game theory because poker is characterized by imperfect information and uncertainty, which are similar to many realistic problems like auctioning, pricing, cyber security, and operations. However, it is not clear that playing an equilibrium policy in multi-player games would be wise so far, and it is infeasible to theoretically validate whether a policy is optimal. Therefore, designing an effective optimal policy learning method has more realistic significance. This paper proposes an optimal policy learning method for multi-player poker games based on Actor-Critic reinforcement learning. Firstly, this paper builds the Actor network to make decisions with imperfect information and the Critic network to evaluate policies with perfect information. Secondly, this paper proposes a novel multi-player poker policy update method: asynchronous policy update algorithm (APU) and dual-network asynchronous policy update algorithm (Dual-APU) for multi-player multi-policy scenarios and multi-player sharing-policy scenarios, respectively. Finally, this paper takes the most popular six-player Texas hold ’em poker to validate the performance of the proposed optimal policy learning method. The experiments demonstrate the policies learned by the proposed methods perform well and gain steadily compared with the existing approaches. In sum, the policy learning methods of imperfect information games based on Actor-Critic reinforcement learning perform well on poker and can be transferred to other imperfect information games. Such training with perfect information and testing with imperfect information models show an effective and explainable approach to learning an approximately optimal policy.

## 1. Introduction

Many games have been solved by artificial intelligence in the last decades, such as checkers [1], chess [2], Go [3,4], etc. However, all these games belong to perfect information games where all the information in the game process can be observed by players. By contrast, it is because poker is characterized by imperfect information and uncertainty, which are similar to many realistic problems, like auctioning, pricing, cyber security, and operations, that poker has been considered a challenging problem in both artificial intelligence and game theory [5].

In game theory, the optimal policy of a game refers to a policy that cannot be exploited by an opponent. On the contrary, the best policy of a game refers to the most profitable policy against certain opponents. The best policy exploits certain opponents by aiming at their weaknesses and habits. A best policy against certain opponents is condemned to be exploited by another policy, such as the best policy against itself. By contrast, the optimal policy ensures not being exploited by an opponent, while not profiting the highest at the same time.

Recently, limit and no-limit poker games of two players have achieved expert levels. For example, [6] solves the heads-up limit hold ’em first, and [7,8] solves heads-up no-limit Texas hold ’em concurrently. These works utilize counterfactual regret minimization to search the game tree, which requires massive computing resources for real-time solving. To enhance the real-time response ability and the decision intelligence, [9,10,11,12,13] utilize reinforcement learning in the simplified variant of two-player poker games. However, two-player poker is identified as a two-player zero-sum game where one player benefits from the other player’s loss. Theoretically, the optimal policy of a two-player zero-sum game has been proved as the Nash equilibrium policy (or approximate ϵ-Nash equilibrium policy in practice). Therefore, the crucial point in solving a two-player zero-sum game is to calculate or approximate the Nash equilibrium policy explicitly.

However, it is not clear that playing an equilibrium policy would be wise in multi-player games so far [14]. Specifically, there may be many or even infinite equilibria in each state of a multi-player game. On the one hand, computing every Nash equilibrium and contrasting their performances are prohibitive for each step. On the other hand, even though each player could compute the equilibrium efficiently, the combination of all the players’ actions could not be an equilibrium if each player computes equilibrium independently, such as in the Lemonade Stand Game [15]. Therefore, since it is infeasible to prove an optimal policy theoretically, designing an effective optimal policy learning method has more realistic significance. 

Multi-agent reinforcement learning (MARL) is a technique introducing reinforcement learning (RL) into the multi-agent system, which brings intelligence to the agents [16]. MARL achieves the cooperation or competition of agents by modeling each player as an RL agent and setting the rewards for their actions. Multi-agent intelligence evolves through the exploration and exploitation of RL agents. RL usually consists of value-based methods and policy-based methods. The valued-based methods approximate value functions with tabular charts and neural networks, typically like DQN [17], Dueling-DQN [18], and Double DQN [19]. The value-based Q-learning will explode when the space dimension increases. The policy-based methods optimize the policy by explicitly minimizing the gradient of the reward sum and action parameters, such as TRPO [20] and PPO [21]. However, the policy-based algorithms are less efficient at optimizing policy due to the policy update based on episodes. Among common RL methods, the Actor-Critic architecture combines the policy-based Actor and the value-based Critic and shows power for many complicated tasks [22]. Additionally, the Actor-Critic architecture is suitable to solve imperfect information problems as the Actor network can be trained to decide with partial observations and the Critic network, providing training aids for the Actor network, can be trained to judge from global observations.

Based on Actor-Critic reinforcement learning, this paper proposes an optimal policy learning method for multi-player poker games. The RL agents can learn from self-play from scratch without any game data or expert skills. This paper will illustrate how Actor-Critic reinforcement learning is applied to multi-player poker games and the according multi-agent policy update methods. The main contributions of this paper are as follows: firstly, this paper builds the Actor network to make decisions with imperfect information and the Critic network to evaluate policies with perfect information; secondly, this paper proposes a novel multi-player poker policy update method: APU and Dual-APU for multi-player multi-policy scenario and multi-player sharing-policy scenario, respectively; finally, this paper takes the most popular six-player Texas hold ’em poker to validate the performance of the proposed optimal policy learning method for multi-player poker games.

The structure of this paper is as follows: firstly, Section 2 introduces the current development of poker algorithms; secondly, Section 3 introduces the modeling of multi-player poker and the fundamentals of multi-agent reinforcement learning; thirdly, Section 4 clarifies the optimal policy learning method based on Actor-Critic reinforcement learning, the network structure for poker learning tasks, and the multi-player policy update method; next, Section 5 designs experiments to evaluate the performance of the proposed methods with existing approaches; and then, Section 6 discusses the results of the preceding experiments; finally, Section 7 concludes this paper.

## 2. Related Work

Taxes hold ’em poker is one of the most popular variants of poker and has been of concern to researchers for a long time. Counterfactual regret minimization (CFR) has been regarded as a method for making decisions in lots of games, based on the virtual traverse and simulation of the decision tree. CFR+, a variant of CFR, is proposed by [6] to relieve the massive memory and computing request of CFR and reports to solve the Heads-up limit hold ’em first. The DeepStack system utilizes a deep neural network to approximate the counterfactual value function and solves heads-up no-limit Texas hold ’em with continual re-solving [7]. Concurrently, the Libratus system proposes game-abstraction, subgame-solving, and self-improver to solve heads-up no-limit poker [8]. This research models poker games as an extensive form of the game tree, traverse all the branches of the game tree, and iterate the policies of game. Additionally, these methods and practices all focus on two-player poker games.

The Pluribus system claims to solve the multi-player poker problem [14]. Pluribus utilizes Monte Carlo CFR (MCCFR) to solve the problem of multi-player optimization: after each round of the game, one player, chosen as a traverser, reviews all the decision nodes in this round, extends the other choices of each node, and calculates the CFR value to optimize the policy of this player. This method assumes each agent knows about the policy of other agents so that they can simulate the counterfactual extended nodes. Additionally, these CFR methods usually require seconds to search on the game tree in real-time response, which is relatively slow.

As deep reinforcement learning solves perfect information games successively, lots of research utilizes reinforcement learning in imperfect games such as poker. Neural fictitious self-play, proposed by [9], combines fictitious self-play and deep reinforcement learning to approximate Nash equilibrium in Limit Texas Hold ’em. Joint-policy correlation architecture into Leduc Poker is proposed by [10], Ref. [11] proposes Actor-Critic architecture for Kuhn and Leduc Poker, Ref. [12] takes Monte Carlo sampling as state evaluation of reinforcement learning for heads-up No-Limit Texas Hold ’em poker, and Ref. [13] utilizes Bayes method to estimate the hand cards of opponents, tuning imperfect information to perfect information, and solves 16-cards Rhode Island Hold ’em poker with reinforcement learning. The aforesaid research that utilizes reinforcement learning into poker only focuses on two-player zero-sum games, and how multi-player poker games learn an optimal policy with reinforcement learning is still scarce until now, which is also the motivation of our work.

## 3. Preliminary

### 3.1. Multi-Player Poker

In this section, we will introduce the fundamental concepts and common terms in multi-player poker for modelling and analyzing. In this paper, we focus on multi-player Texas Hold ’em poker.

**Figure 1:**
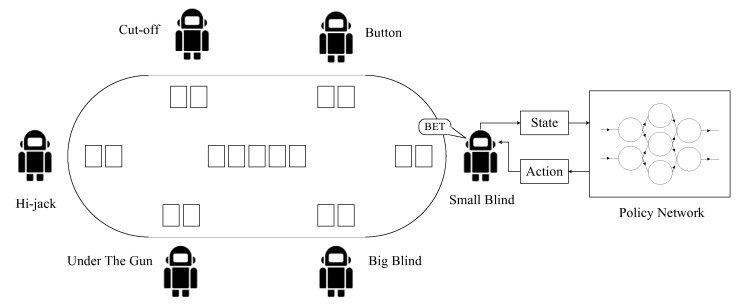
The modelling of multi-player Taxes hold ’em poker: the positions, hole cards, community cards, and the policies of players.

Multi-player Texas Hold ’em poker is usually participated by 2–12 players and the most commonly played format is six-player poker. There are 52 cards in a deck of Texas Hold ’em. At the beginning of a board, each player is dealt two hole cards, which only can be seen by themselves. Meanwhile, five community cards are dealt onto the board, faced down initially. Each board of poker designates a player as Button, and players to the left of Button are Small Blind, Big Blind, and Under The Gun, as shown in Figure 1. Big Blind and Small Blind are forced to wager initially, where Big Blind usually bets twice as much as Small Blind does.

There are four rounds in a board of Taxes hold ’em: Pre-flop, Flop, Turn, and River. In the Pre-flop round, players declare their intentions from Under The Gun sequentially in clockwise order. The declaration starts from Small Blind in the Flop, Turn, and River rounds. The intentions of poker players usually include three types:

Fold: Fold represents the player forfeiting this board out of the following rounds and declarations. The chips already wagered into the pot will not be returned and his hole cards are faced down without showing.

Call: Call means that the player would wager chips up to the most amount of chips wagered by other players currently. If the player himself has already been the one who wagered most, this intention is also called Check.

Bet: Bet means that the player would wager more chips into the pot, which will aggrandize the most amount of chips wagered. If a player bets before and this player chooses to bet, this intention is also called Raise.

In each round, players declare their intentions in a clockwise direction until the last bet is called by all players unless they have folded. The flow of rounds is shown in Algorithm A1. Apparently, such declarations result in the uncertain length of each round. Moreover, in each new round, part of the community cards is turned over: the first three cards in Flop, the fourth card in Turn, and the fifth card in River.

As for the rules of winning, if there is only one player who has not folded and others all folded in any round, this player will win explicitly. If there is more than one player remaining after four rounds, these players are confirmed as winning or losing through Showdown. In Showdown, each player should choose five cards from seven cards, two hole cards, and five community cards, as they draw cards. The draw cards determine which player wins this pot, and the categories, and ranks of which are shown in Table A1. The winning player will gain all the chips in the pot or share the pot with all winners.

In multi-player poker, we classify the information as follows. The personal information includes the hole cards with ranks and suits. The public static information includes the community cards, the chips each player has, and the amount of chips each player has wagered into the pot. The public dynamic information includes all the action sequences of all players on this board. The abovementioned three kinds of information are the observations of reinforcement learning agents, upon which the agents base their actions. Section 4.3 will clarify the observation modelling in detail.

### 3.2. Multi-Agent Decision Environment

In this paper, a multi-agent decision environment includes N agents, where i denotes the ith agent and −i denotes other agents except i. In imperfect information games or partial observable stochastic games, the world state w denotes the global information of the environment. As for the agent i, its observation is $O_{i}=O_{i}\left(w\right)$ and its action $a_{i}=\pi_{i}\left(O_{i}\right)$ is based on its policy $\pi_{i}$. Furthermore, in simultaneous games (A simultaneous game or static game is a game where each player chooses their action without knowledge of the actions chosen by other players.), the new world state $w^{\prime }=W\left(w,a\right)$ is dependent on the original world state and the joint action $a=\left(a_{1},a_{2},\ldots ,a_{N}\right)$. However, in poker, such a typical sequential game (A sequential game is a game where one player chooses their action before the others choose theirs.), if it is the ith agent’s turn to decide, the new world state $w^{\prime }=W\left(w,a_{i}\right)$ is dependent on the original world state and this agent’s action. Since this paper specifically aims at multi-player poker, which belongs to sequential games, the following paper will only discuss the methods and practice on sequential games.

The history (also called trajectory) of a game is the sequence of a multi-agent’s actions and world states, $h=\left(w_{0},a_{1}^{1},w_{1}^{1},a_{2}^{1},w_{2}^{1},\ldots ,\;a_{N}^{1},w_{N}^{1},a_{1}^{2},w_{1}^{2},\ldots \right)$, where $a_{i}^{j}$ denotes the jth action of the agent i, $w_{i}^{j}$ denotes the world state after the agent i executes its jth action $a_{i}^{j}$, and $w_{0}$ denotes the initial state. Since there is no stochastic influence from the players’ actions in poker, the history of actions $h_{a}=\left(w_{0},a_{1}^{1},a_{2}^{1},\ldots ,\;a_{N}^{1},a_{1}^{2},\ldots \right)$ has the same information as the history h. Explicitly, the history of actions $h_{a}$ is a datum of undefined length. We refer to it as public dynamic information, mentioned in Section 3.1, which inspired the design of the network structure in Section 4.2.

## 4. Methods

### 4.1. Multi-Player Poker Policy Learning via Actor-Critic RL

**Figure 2:**
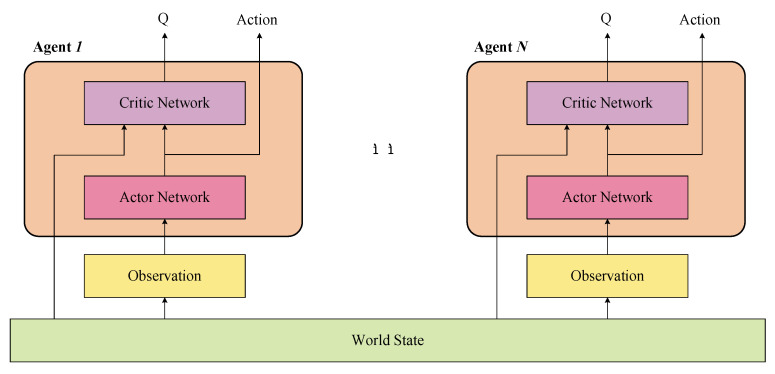
Actor-Critic reinforcement learning architecture of N agents.

In this paper, the multi-agent reinforcement learning for multi-player poker policy learning is based on one of Actor-Critic (AC) reinforcement learning architecture, and the Deep Deterministic Policy Gradient [22]. The Actor and Critic part of multi-agents are shown in Figure 2. On the one hand, given the world state w, the Actor part of the agent i inputs its observation $O_{i}\left(w\right)$ and outputs its decision action $a_{i}=\pi_{i}\left(O_{i}\right)$. On the other hand, the Critic part of the agent i could obtain the world state (includes information of other agents) and its action $a_{i}$ from its Actor part, and outputs $Q_{i}\left(w,a_{i}\right)$. $Q_{i}\left(w,a_{i}\right)$ represents the evaluated value of choosing action $a_{i}$ under the world state w.

The merit of such Actor-Critic architecture is that multi-agents can train with perfect information and execute with imperfect information. In the training procedure, the Critic part, with global information, is brought in to direct the Actor part training its policy. While, in the executing procedure, the Actor part could take its action on the partial observations. Therefore, the Critic provides training assists for the Actor, and after the training of the Actor is finished, the Actor could make the decision independently. In this way, the well-trained Actor part can output the policy under imperfect information.

In this paper, we conduct experiments under two typical scenarios. In the stage of multi-player poker, each player is supposed to maintain an Actor-Critic architecture. Namely, each player makes a decision based on its own Actor part (policy), which is directed by its own Critic part (policy evaluation), denoted as a multi-agent multi-policy scenario. Moreover, since all the poker players, or the RL agents, have the same functions and structures, multi-player pokers could share one set of Actor-Critic architecture for decision. This is the multi-agent sharing-policy scenario, contrary to the above-mentioned multi-agent multi-policy scenario.

### 4.2. Network Structure of Reinforcement Learning

**Figure 3:**
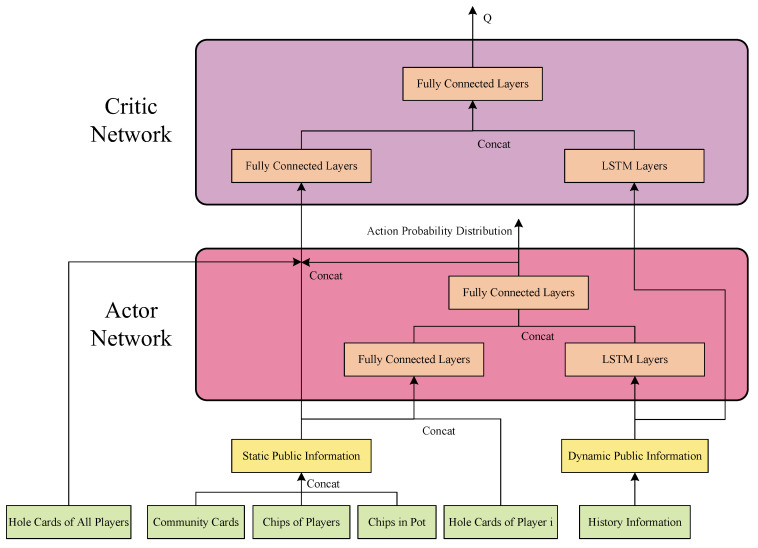
Network structure of Actor-Critic in multi-player poker.

In this section, we will clarify the architecture of multi-player poker policy learning and the network structure of Actor-Critic, as shown in Figure 3. For the multi-player poker task, this paper divides the observations of agents into public information and personal information. The personal information consists of the ranks and suits of their own hole cards. The public information involves the suits and ranks of public cards, the chips of all the players, the chips each player has wagered (or the amount of pot), and last but not least, the history of the players’ actions. Specifically, the history of the players’ actions keeps accumulating during the poker game and it is apparently of undefined length, which is denoted as public dynamic information. Other public information has defined length and is denoted as static public information. 

For the Actor network, it aims at calculating the action probability distribution, based on the observation of the reinforcement learning agent. When the static public information and personal information are received, which are of definite length, the Actor extracts features with a network of full-connected layers. The Actor employs Long Short-Term Memory (LSTM) layers to extract features from the dynamic public information, which involves sequential implication and of indefinite length. Then, full-connected layers synthesize the outputs of these two feature extractions and export the action probability distribution in a certain format.

For the Critic network, the function of the Critic part is to evaluate whether it is wise to make this decision under the state of this agent. Therefore, the Critic network requires all personal information of all players and public information. Similar to Actor, the Critic network builds fully-connected layers to extract features of personal information and static public information and LSTM layers to model the history of actions. Finally, $Q_{i}(O_{i},a_{i}|w)$ is outputted after the full-connected layers.

In the network training, the optimization direction of the Actor network is to maximize $Q_{i}\left(w,a\right)$. However, it is prohibitive to obtain a precise $Q_{i}$ and the output of the Critic network $\hat{Q_{i}}\left(w,a\right)$, the estimation of $Q_{i}$, is taken as optimization substitution. The Critic network is to minimize the deviation between the prediction estimation $\hat{Q_{i}}\left(w,a\right)$ and the true value $Q_{i}\left(w,a\right)$. After iteratively calculating, given the reward r of this action, we have $Q_{i}\left(w,a\right)=E\left[r+Q_{i}\left(w{}^{\prime },a{}^{\prime }\right)]\approx E[r+\hat{Q_{i}}\left(w{}^{\prime },a{}^{\prime }\right)\right]$. Hence, the Critic network is to minimize loss $L={\left[\hat{Q_{i}}\left(w,a\right)-\left(r+\hat{Q_{i}}\left(w{}^{\prime },a{}^{\prime }\right)\right)\right]}^{2}$. Such optimization of Q value is the same as deep Q-learning, where Q network and target network could stabilize optimization [23]. The reward modeling will be introduced in the next section.

### 4.3. Observation and Reward Modeling

For each agent i, the information state infostate, also called an Action-Observation History (AOH), is the sequence of its observations and actions $s_{i}=\left(O_{i}^{1}ˉ,a_{i}^{1},\;O_{i}^{1},\ldots ,O_{i}^{2}ˉ,\;a_{i}^{2},O_{i}^{2},\ldots \right)$. Specifically, $\;O_{i}^{j}ˉ$ denotes the observation of agent i before its action j, where we have $a_{i}^{j}=\pi_{i}\left(\;O_{i}^{j}ˉ\;\right)$. $\;O_{i}^{j}$ denotes the observation of agent i after its action j, i.e., $\;O_{i}^{j}=O\left(w_{i}^{j}\right)$.

For the reinforcement learning of the agent i, the agent i obtains its observation $O_{i}ˉ\;$ at the world state w and chooses its action $a_{i}=\pi_{i}\left(O_{i}ˉ\;\right)$ according to its policy $\pi_{i}$. After that, the world state transits to $w_{i}$ and the environment feedbacks the reward $r_{i}=R\left(w,\;a_{i}\right)$, based on the world state before and the action. The reward of reinforcement learning agents affects the performance of learned agents and we set the reward function of multi-player poker task as following:

(1) $$r_{i}=\left\{\begin{array}{c} 0\;\;\;\;\;\;\;\;\;\;\;\;\;\;\;\;\;\;\;\;if\;Fold\\ -the\;chips\;required\;commiting\;\;if\;Call/Bet\\ {\left(\frac{the\;pot}{the\;number\;of\;winners}\right)}^{2}\;\;\;\;\;\;if\;Win\; \end{array}\right.$$

This reward function ensures that when agents wager chips, they get equal punishment. A squared encouragement is given when they win the pot. This would help the agents wager chips to participate in this board or appeal other agents to wager more, where the final winning gain will compensate for the early investment. The whole target of reinforcement learning agents is to obtain higher $\sum_{t=0}\gamma^{t}r_{i}^{t}$, where γ denotes attenuation term. If γ is closer to 1, the agent is more farsighted and more nearsighted if γ is closer to 0. In the multi-player poker task, we believe every decision is equally significant, therefore, we take γ=1.

### 4.4. Experience Modeling

After each decision, reinforcement learning could accumulate the decision experience for off-line learning. The experience of reinforcement learning agents usually includes the original state (or observation), action, new state (or observation), reward, whether the task is finished, etc. [22]. In multi-agent reinforcement learning for simultaneous games, the state transits before and after the multi-agent joint action. Therefore, experience in simultaneous games includes the state before and after the joint action, the action of one agent or the joint action, the reward, whether they have finished or not, etc. However, in sequential games, the state transits when each agent takes their actions sequentially. There is no explicit principle to choose a new state (or observation) for experience in multi-agent sequential games. Here we show the process of multi-agent sequential games as follows.

For the action j and the action j + 1 of the agent i in a game process:

(2) $$(w_{i-1}^{j},\;O_{i}^{j}ˉ\;\;\;)\overset{a_{i}^{j}}{\to }\left(w_{i}^{j},\;\;O_{i}^{j}\right)\;\overset{a_{i+1}^{j}}{\to }\ldots \overset{\;\;\;a_{N}^{j}\;\;\;}{\to }\;\overset{a_{1}^{j+1}}{\to }\ldots \overset{a_{i-1}^{j+1}}{\to }\left(w_{-i}^{k+1},\;\;\;O_{i}^{j+1}ˉ\;\;\right)\overset{a_{i}^{j+1}}{\to }$$

The original observation of the action $a_{i}^{j}$ is definitely $\;O_{i}^{j}ˉ$, with reward $\;r_{i}^{j}$. Yet, either $\;O_{i}^{j}$ or $\;O_{i}^{j+1}ˉ\;$ could be the new observation of its action, to some degree. On the one hand, taking $\;O_{i}^{j}$ as the new observation of this experience means this experience observes the state right before and after this action. In this way, the experience contains no decisions of other agents and is kind of stable for the agent. On the other hand, the agent observes before the moment it makes two adjacent actions and therefore the experience contains the actions of other agents, which records the aftereffect of this action. We conclude the two kinds of experience as:

Short experience $e_{short}=\left(\;O_{i}^{j}ˉ\;,\;a_{i}^{j},\;O_{i}^{j},\;r_{i}^{j}\right)$. The experience is collected before and after the action $a_{i}^{j}$.

Long experience $e_{long}=\left(\;O_{i}^{j}ˉ\;,\;a_{i}^{j},\;\;O_{i}^{j+1}ˉ\;,\;r_{i}^{j}\right)$. The experience is collected before the jth action and the $\left(j+1\right)$th action.

The authors in [9] describe the experience as, quote, experience in the form of transition tuples, $\left(s_{t},\;a_{t},\;r_{t+1},\;s_{t+1}\right)$, where $s_{t}$ is the state at time t, $a_{t}$ is the action chosen in that state, $r_{t+1}$ the reward received thereafter, and $s_{t+1}$ the next state that the agent transitioned to. Here this experience seems to be a short experience. Yet in the following description, quote, the sequence of his information states and actions, $s_{i}^{1},a_{i}^{1},s_{i}^{2},a_{i}^{1},\ldots ,\;s_{i}^{t}$, is more likely to be long experience. How the experience is constructed for agents has great influence on the performance in multi-player games. Since the early research has not pointed a definite way, we take long experience in this paper, because the experience could be iterated, i.e., the new observation of this piece of experience will be the original observation of the next one.

### 4.5. Multi-Player Poker Policy Update

In the process of multi-player policy learning, how to update the policies of multi-agent is another crucial problem. The most explicit way is to maintain N reinforcement learning networks for each agent, and they learn from each other in the random interaction. However, that agents learn independently and that multi-agents choose their own optimization directions will result in higher indeterminacy of other agents’ policies observed. Therefore, each agent finds it hard to identify the best policy gradient direction, and the whole crowd may fail to increase their intelligence. Such a problem is not notable in two-player games, since the indeterminacy each agent observed is only from the other agent so the indeterminacy space is explorable. However, taking the six-player Texas hold ’em poker as an example, the dynamic public information is informed by other agents’ actions and the state space is exponential to the number of agents and their policy types. When every agent keeps changing its policy, therefore giving different actions in similar circumstances, the difficulty of reinforcement learning convergence increases remarkably.

To overcome this problem, this paper proposes a multi-agent asynchronous policy update algorithm (APU) for multi-player poker, shown in Algorithm 1. In the learning procedure of multi-agent reinforcement learning, each agent maintains and optimizes its own policy. However, different from traditional policy optimizing parallelly, the APU chooses one reinforcement learning agent to learn and optimize in a period (AGENT_PERIOD). Every agent is sequentially chosen to optimize its policy. In the APU method, only one agent is optimizing its policy once a time while others remain static, which makes the optimizing direction of this agent more explicit.
**Algorithm 1:** Multi-Agent Asynchronous Policy Update Algorithm (APU) for Multi-player Poker1Initialize Actor-Critic network for each player2**for** *episode* ← 1 **to** EPISODE3 **for** *button* ← 1 **to** $N_{player}$
4   Play a board of Texas hold ’em as **Algorithm A1**5 **end for**6 **if**
*episode*
**mod** AGENT_PERIOD **==0:**7   shift to another *training_agent* sequentially8 **end for**9 **if**
*episode*
**mod** TRAINING_PERIOD **==0:**10   update Actor-Critic network of *training_agent*11 **end if**12**end for**

On the other hand, considering the homogeneity of multi-agents in the multi-player poker games, it is unnecessary for multi-agents to learn their policies repetitively. The authors of [24] have shown parameter sharing among cooperative agents to improve training efficiency. Multi-agents sharing one set of network parameters could decrease the memory space and training cost. However, when all the agents share one policy, the policy network may converge to a local minimum rather than the global optimal policy. In this regard, [25] proposes fictitious self-play and proves the self-play method could reach the Nash equilibrium in two-player zero-sum games. Inspired by this idea, this paper proposes the dual-network multi-agent asynchronous policy update algorithm (Dual-APU) for multi-player poker, shown in Algorithm 2.
**Algorithm 2:** Dual-Network Multi-Agent Asynchronous Policy Update Algorithm (Dual-APU) for Multi-Player Poker1Initialize an online Actor-Critic network2Initialize a target Actor-Critic network3**for** *episode* ← 0 **to** EPISODE4 **for** *button* ← 1 **to** $N_{player}$
5   Play a board of Texas hold ’em as **Algorithm A1,** where player 1 acts via online network and other players act via target network6 **end for**7 **if**
*episode*
**mod** UPDATE_PERIOD **==0:**8   **update** online Actor-Critic network to target Actor-Critic network9 **end for**10 **if**
*episode*
**mod** TRAINING_PERIOD **==0:**11   update online Actor-Critic network12 **end if**13**end for**

In order to avoid the sharing network converging to a local minimum, the Dual-APU is proposed. Generally, the Dual-APU maintains two sets of policy networks: the online network and the target network. Specifically, the online network serves a certain agent and optimizes its policy based on the experience of this agent fighting with opponents of the target network. The target network is called by all except one of the multi-agents to choose actions and is not optimized by the system. After a period (UPDATE_PERIOD) of optimization, the parameter of the online network is updated to the target network, so that the policies of other agents are upgraded as well. Moreover, due to the homogeneity of agents in multi-player poker tasks, the Dual-APU takes agent 1 as the one who uses the online network to make decisions (line 5 in Dual-APU), without a loss of generality. This training process ensures the other five agents make decisions with the updated policy while unchanged and one agent keeps learning and optimizing their policy during the interactions and competitions with these opponents. Every once in a while, the newly learned policy will update the policy of the opponents and therefore the online network and the target network keep improving together.

## 5. Results

In this section, the policies of multi-player poker learned from multi-agent reinforcement learning will be compared with existing policies or policies learned in different settings. Since multi-player poker is a typical multi-agent game problem, we take six-player Texas hold ’em to evaluate the performances. To contrast explicitly any two policies, the experiments in this section obey the following regulations.

(1) 1 versus 5 games. When contrasting the performance between two policies, one player employs the policy to be evaluated as the experimental group and five other players employ the other policy as the control group;
(2) Random positions. The six players are distributed to random positions at the beginning of each board. On each board, every player will be designated as Button once by turns;
(3) The mean gains of 1000 boards. The six players play 1000 boards (6000 hands) and the mean gains are calculated. Here we calculate the small bets per hand (sb/h), where the total number of small bets won or lost is divided by the total hands played.

In multi-player Texas hold ’em poker, the performances or gains of policies are highly relevant to the policies composition and the positions of players. Therefore, on the one hand, that one player from the experimental group and six players from the control group is an approach to evaluate the experimental policy against the other policy independently, which avoids collusion between two players of the same policy. On the other hand, random positions of the 1000 boards and that all the players play as Button by turn decreases the influence of players’ positions and the positions relevant to the Button. Finally, the influence of randomness from Texas hold ’em poker is analyzed statistically in the mean gains of the 1000 boards.

The experiments are deployed on the Ubuntu 18.04 (Linux version 5.4.0-58-generic), with CPU: Intel(R) Xeon(R) CPU E5-2620 v4 @ 2.10GHz and GPU: NVIDIA GeForce RTX 2080. The codes are implemented by Python 3.7.4 and TensorFlow 2.3.1.

### 5.1. Contrast with Existing Policies

In this section, we contrast the poker policy learned from multi-agent reinforcement learning with existing policies. The existing policies are as follows:

(1) Policy based on Sklansky hand ranking

David Sklansky classified the hands of poker into different categories according to his poker experience and the winning rate of all-in in the Pre-flop round [26]. These categories are arranged with ranking in Table A2. With no regard for bluff, the policy tends to bet or call if the hand is strong while it tends to call or fold if the hand is weak.

(2) Policy based on Bill Chen hand estimation

Noted Texas poker player Bill Chen provided Chen Formula to estimate the strength of hands [27]. In this way, the estimation comprehends the suits, ranks, pairs or not, etc., to quantify the scores of hands. Similarly, this policy tends to bet or call if the score is high. The Bill Chen hand estimation is shown in Algorithm A2.

(3) Policy based on rules

The above-mentioned two policies are based on the hands of players, with no regard for the coupling relationship of hole cards and community cards. Therefore, this paper utilizes a policy based on self-made rules, according to the same rank, same suit, or straight of hole cards and community cards to make decisions. Generally, this policy still tends to bet if the hand strength is strong.

(4) Policy based on Monte Carlo Tree Search

Monte Carlo Tree Search (MCTS) is a widespread game tree search method [28]. MCTS models a game process as a game tree according to the decision sequence. When MCTS extends a new node of the game tree, the MCTS simulates the node and its successor nodes based on the policies of all players. Simulation demonstrates the value of this node, and the value is updated to the preorder pathways. Additionally, the MCTS utilizes the Monte Carlo ideology to judge the decision value of every node in the game tree, based on which the MCTS policy can make decisions.

This section will contrast the policy learned from the APU and Dual-APU. Such two policies are contrasted with the above-mentioned policies, respectively. The gains of 1000-board games are as Table 1.

### 5.2. The Effect of Asynchronous Policy Update

As reported in Section 4.5, this paper proposes the asynchronous policy update methods, i.e., Dual-APU and Dual-APU, applied to the multi-player multi-policy scenario and the multi-player sharing-policy scenario, respectively. To validate the effect of the asynchronous policy update methods, we design the following contrast experiments.

(1) Multi-player multi-policy scenario

In the multi-player multi-policy scenario, the most explicit policy update way is that each reinforcement learning agent maintains a policy network, accumulates its experience into an experience set, and trains its own policy network based on its experience. Such an independent way gets the policy denoted as APU-Ctrl, the process of which is shown in Algorithm A3.

(2) Multi-player sharing-policy scenario

In the multi-player sharing-policy scenario, multi-agents share one policy network, accumulate experience into the sharing experience set, and train the sharing policy network based on the sharing experience. When it is time for an agent to act, the agent could call the sharing policy network to make decisions. The process of the sharing policy is shown in Algorithm A4, upon which the policy is denoted Dual-APU-Ctrl.

In this section, we take two basic learning methods (APU-Ctrl/Dual-APU-Ctrl) as the control group and APU/Dual-APU as the experimental group to conduct contrast validation. The APU/Dual-APU are contrasted with APU-Ctrl/Dual-APU-Ctrl, respectively. The results are shown in Table 2.

Additionally, the APU-Ctrl and Dual-APU-Ctrl are contrasted with the existing policies mentioned in Section 5.1. The results are shown in Table 3. In conclusion, the multi-agent reinforcement learning methods perform well in multi-player poker games and the proposed asynchronous policy update methods could enhance the ability of learning policies.

### 5.3. The Effect of Dynamic Public Information

In the multi-player poker policy learning method based on Actor-Critic architecture, the network builds specific layers and structures to perceive and analyze the information of multi-player poker. Specifically, in Section 4.2 (or Figure 3), we utilize the full-connected layers to extract features from the information of definite length, and LSTM layers to extract features from the dynamic public information (history of actions). To validate the distinct processes for different input information, we design the ablation experiments to demonstrate the effect of dynamic public information. The two benchmarks are described as follows:

(1) None of dynamic public information (None)

Explicitly, this setting keeps the parameters of LSTM layers in the Actor and Critic network as zero. Then the output of the dynamic public information after LSTM layers will be a series of zeros. This will certainly shield the dynamic public information and eliminate the effect of the dynamic public information.

(2) Full-connected (FC)

In this setting, we replace the LSTM layers in the Actor and Critic network as full-connected layers. In this way, the network structure retains the perception and the feature extraction ability of the dynamic public information, only to use full-connected layers instead of LSTM. This experiment could validate the feature extraction ability of LSTM for the dynamic public information.

This section takes these two settings into the APU and Dual-APU and contrasts the gains to validate the effects of different network structures. The results are shown in Table 4 and Table 5.

## 6. Discussion

### 6.1. RL Poker Policy Performs Well

In experiment 5.1, the proposed multi-player poker policy learned by multi-agent reinforcement learning (the APU and Dual-APU) performs well in the games against existing policies. Additionally, the learned policies could gain steadily against policy based on hands (Sklansky, Bill Chen), policy based on rules, and MCTS policy. Therefore, it is demonstrated that the policy learned by RL approximates the optimal policy in the statistical significance.

Specifically, the multi-player poker policy learning methods based on RL aim at learning a sufficiently approximate optimal policy of multi-player poker. The optimal policy denotes the policy not being exploited by facing any opponents. On the contrary, the best policy denotes the policy profits the highest against certain opponents. Therefore, the best policy is relevant to opponents and must counter the drawback and styles of the opponents. Meanwhile, since the best policy can exploit certain opponents, it will definitely be exploited by the best policy against itself. Hence, the best policy has quite a little generalization, while the optimal policy ensures not being exploited all the time though not profiting most in some time either.

Aiming at the optimal policy, the multi-player poker policy learning method utilizes only self-play to train themselves. Six players’ policies or the sharing poker policy of Texas hold ’em poker are all initialized randomly and learned from scratch. The policies are optimized gradually in the self-play poker games and finally reach the convergence of the policy network. To validate whether the policy learned plays well when the policy network converges, the experiments of learned policies against other policies are conducted, which verify the multi-player poker policy by RL performs well and stably.

### 6.2. Multi-Agent Asynchronous Policy Update Performs Stably

In Section 5.2, we contrast the policy learned by the proposed multi-agent asynchronous policy update algorithm (the APU and Dual-APU) with the policy learned by independent optimization. The results demonstrate that the APU and Dual-APU perform well and stably.

**Figure 4:**
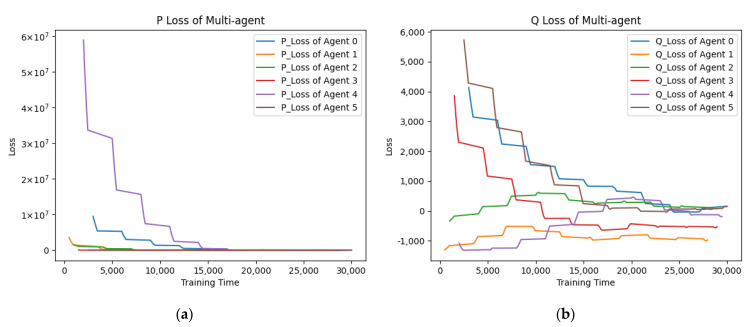
The loss curves of the Actor and Critic network in the asynchronous policy update algorithm (APU). (**a**) The P Loss represents the loss of the Actor network (left). (**b**) The Q Loss represents the loss of Critic network (right).

In the multi-player multi-policy scenario, the proposed APU has superiority over independent optimization. This is because when multi-agent optimizes their own policy, each agent calculates their optimization directions according to their own experience, which results in the police vibrations of all agents. Moreover, if an agent has a poor policy, all the other agents will keep exploiting it and cannot learn preferable policies, which will affect the total optimization performances. However, in the APU, when each agent optimizes its policy, the policies of other agents remain invariable, as shown in Figure 4. Then only one agent modifies its policy against its opponents at any time. This method ensures the crowd intelligence promoting stably.

**Figure 5:**
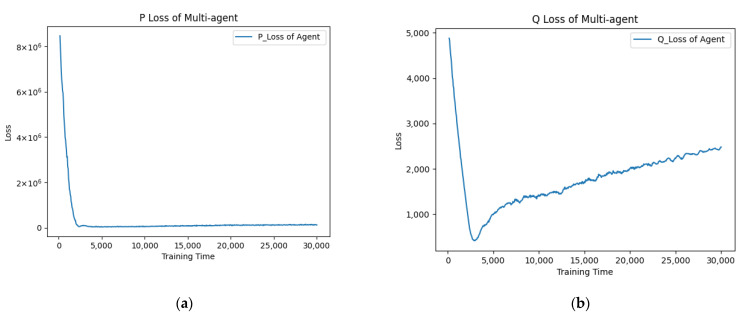
The loss curves of the Actor and Critic network in the dual-network asynchronous policy update algorithm (Dual-APU). (**a**) The P Loss represents the loss of the Actor network. (**b**) The Q Loss represents the loss of the Critic network.

In the multi-player sharing-policy scenario, the Dual-APU has more promotion than common policy sharing. In common policy sharing, all the agents accumulate their experience into sharing set, which is used to optimize the sharing policy network. Such an approach enlarges the available experience set, which will enhance the generalization of the sharing policy. On the contrary, the Dual-APU, based on the self-play ideology, maintains one agent optimizing its policy (online policy) while other agents make a decision according to the previous policy (target policy), as shown in Figure 5. After a period of time, the online policy will update to the target policy. In such an approach, the experience for online policy training is only from one agent and this constrains the optimization speed in part. Therefore, a slight improvement has been achieved for the multi-player sharing-policy scenario.

### 6.3. Dynamic Public Information Is Helpful

In experiment 5.3, we validate the feature extraction effect of the LSTM layers. The results demonstrate that the LSTM structure has apparent improvements for fully-connected structures, both in multi-player multi-policy and multi-player sharing-policy scenarios. This proves that the LSTM network structure is more powerful at extracting features of dynamic public information, a kind of temporal information.

Further, the LSTM and the fully-connected structure perform better than none of the dynamic public information, which proves the crucial significance of the dynamic public information in multi-player poker games. The dynamic public information includes the history of all players’ actions on this board and is valuable for reference when agents act.

Based on the above-mentioned ablation experiments, we draw the conclusion that the dynamic public information is helpful for the multi-player poker games and the LSTM network structure has positive effects on extracting features from the dynamic public information.

### 6.4. Real-Time Response in Poker Games

The purpose of the multi-player poker policy method based on multi-agent reinforcement learning is to generate an optimal multi-player poker policy. Therefore, the ability of the real-time response is necessary for such a policy system. In the test procedure after training, the proposed APU and Dual-APU are able to make decisions in 7.3 ms on average, which is definitely a real-time level.

Compared with the rapid response in the test procedure, the APU and Dual-APU spend computing resources on the training of multi-player poker policies. In the training procedure, the network is trained for 20,000 episodes with each player being Button once in each episode, and the APU consumes 12.5 h and the Dual-APU consumes 7.6 h. Generally, such methods consume lots of computing resources to optimize the policy during the training procedure, while the testing and calling are rapidly responded to without a large amount of consumption. This ideology that separates the policy learning and policy application meet the demand of policy response in real-time.

## 7. Conclusions

Poker has been considered a challenging problem in both artificial intelligence and game theory because poker is characterized by imperfect information and uncertainty, which are similar to many realistic problems like auction, pricing, cyber security, and operations. However, it is not clear that playing an equilibrium policy in multi-player games would be wise so far. Since it is impossible to prove an optimal policy theoretically, designing an effective optimal policy learning method has more realistic significance. This paper proposes an optimal policy learning method for multi-player poker games, based on Actor-Critic reinforcement learning. The reinforcement learning of multi-agents leads the policy evolution procedure to self-play from scratch without any game data or expert skills.

This paper proposes an optimal policy learning method for multi-player poker games, based on Actor-Critic architecture. Firstly, this paper builds the Actor network to make decisions with imperfect information and the Critic network to evaluate policies with perfect information, where the Actor-Critic architecture trains with perfect information and executes with imperfect information. Secondly, this paper designs network structures for Actor-Critic reinforcement learning, the full-connected layers, and LSTM layers to extract features from observations of definite length and the history of actions, respectively. Finally, this paper proposes the multi-player poker policy update methods: asynchronous policy update algorithm (APU) and dual-network asynchronous policy update algorithm (Dual-APU) for multi-player multi-policy scenarios and multi-player sharing-policy scenarios. These multi-agent policy update methods solve the problem of mutual interference in independent optimizations.

This paper takes the most popular six-player Texas hold ’em poker to validate the performance of the proposed optimal policy learning method for multi-player games. The experiments demonstrate the policies learned by the proposed methods perform well and gain steadily with the policy based on hand strength, the policy based on rules, and the MCTS policy. The contrasting experiments validate that the proposed APU and Dual-APU have more stable training procedures and better performance than independent optimizations. Further, the ablation experiments show that the network structure designed for Actor-Critic is effective. Finally, the multi-player poker policy learned by multi-agent reinforcement learning can respond in real-time.

Last but not least, the policy learning methods of imperfect information games based on Actor-Critic reinforcement learning can be transferred to other imperfect information games more than poker. Such training with perfect information and testing with an imperfect information model shows an effective and explainable approach to learning an approximately optimal policy. Meanwhile, the current work cannot exploit certain opponents with specific playing styles and improve the profit against an acquainted opponent. This will also be our future work in the next stage.

## Figures and Tables

**Table 1:** The contrast gains of APU and Dual-APU against the existing policies.

	Sklansky	Bill Chen	Rule-Based	MCTS
APU	4.083\−4.083	0.957\−0.957	3.265\−3.265	3.895\−3.895
Dual-APU	1.833\−1.833	0.542\−0.542	2.167\−2.167	3.376\−3.376

Note: The X\Y in the table represents the gain of the row policy is X and the gain of the column policy is Y. The units in the table are all sb/h (small bets per hand).

**Table 2:** Contrast gains of APU and Dual-APU against the control group, respectively.

	APU\APU-Ctrl	Dual-APU\Dual-APU-Ctrl
Gain	0.031\−0.031	0.003\−0.003

Note: The units in the table are all sb/h (small bets per hand).

**Table 3:** The contrast gains of APU-Ctrl and Dual-APU-Ctrl and the existing policies.

	Sklansky	Bill Chen	Rule-Based	MCTS
APU-Ctrl	2.275\−2.275	0.676\−0.676	2.726\−2.726	1.303\−1.303
Dual-APU-Ctrl	1.981\−1.981	0.607\−0.607	2.882\−2.882	1.804\−1.804

Note: The X\Y in the table represents the gain of the row policy is X and the gain of the column policy is Y. The units in the table are all sb/h (small bets per hand).

**Table 4:** The gain contrast of different settings in APU.

	APU-None	APU-FC
APU-1	9.728\−9.728	1.251\−1.251

Note: The units in the table are all sb/h (small bets per hand).

**Table 5:** The gain contrast of different settings in Dual-APU.

	Dual-APU-None	Dual-APU-FC
Dual-APU	0.918\−0.918	0.672\−0.672

Note: The units in the table are all sb/h (small bets per hand).

## Data Availability

Not applicable.

## Appendix A

**Algorithm A1:** Texas Hold ’Em Process	
1	Choose a player as Button
2	**for** *round* in [‘Pre-Flop’, ‘Flop’, ‘Turn’, ‘River’]
3	**if** *round*=‘Pre-Flop’
4	*head* ← *button*+3
5	**else**
6	*head* ← *button*+1
7	**end if**
8	*tail* ← *head*+$N_{player}$-1
9	**while** head<=tail
10	*player* ← *head* **mod** $N_{player}$
11	**if** *player* has folded
12	*head* ← *head+1*
13	**continue**
14	**end if**
15	*player* chooses his *action*
16	**if** *action* is BET
17	*tail* ← *head*+$N_{player}$-1
18	**end if**
19	*head* ← *head+1*
20	**end while**
16	**end for**

**Table A1:** Hand-ranking categories of Texas hold ’em poker.

Hand-Ranking Categories	Example
Straight flush	Q♥ J♥ 10♥ 9♥ 8♥
Four of a kind	9♣ 9♠ 9♦ 9♥ J♥
Full house	3♣ 3♠ 3♦ 6♣ 6♥
Flush	K♣ 10♣ 7♣ 6♣ 4♣
Straight	7♣ 6♠ 5♠ 4♥ 3♥
Three of a kind	2♦ 2♠ 2♣ K♠ 6♥
Two pair	4♥ 4♠ K♠ 10♦ 5♠
One pair	6♦ 6♥ Q♥ J♠ 2♣
High card	Q♥ 10♦ 7♠ 5♠ 2♥

**Table A2:** The hand range categories by David Sklansky.

Hand Range	Category
Very high(3%)	AA-JJ and AK
Tight(5%)	AA-99 and AK-AQ
Average(10%)	AA-77, AK-AT, and KQ
Loose(25%)	AA-22, AK-A5 Any two cards T or higher(e.g., QT), and k9s-T9s
Very loose(50%)	AA-22, AK-A2, Any cards 7 or higher(e.g., T7), K6-K4, and K3s-K2s
Any two(100%)	Everything

**Algorithm A2:** Chen Formula for Hand Rankings	
1	Based on the highest card, assign points as follows: Ace = 10 points, K = 8 points, Q = 7 points, J = 6 points. 10 through 2, half of face value (10 = 5 points, 9 = 4.5 points, etc.)
2	For pairs, multiply the points by 2 (AA = 20, KK = 16, etc.), with a minimum of 5 points for any pair. 55 is given an extra point (i.e., 6).
3	Add 2 points for suited cards.
4	Subtract 1 point for 1 gappers (AQ, J9) 2 points for 2 gappers (J8, AJ). 4 points for 3 gappers (J7, 73). 5 points for larger gappers, including A2 A3 A4
5	Add an extra point if connected or 1-gap and your highest card is lower than Q

**Algorithm A3: APU-Ctrl:** Independent Policy Update Algorithm	
1	Initialize Actor-Critic network for each player
2	**for** *episode* ← 1 **to** EPISODE
3	**for** *button* ← 1 **to** $N_{player}$
4	Play a board of Texas hold ’em as **Algorithm A1**
5	**end for**
6	**if** *episode* **mod** TRAINING_PERIOD **==0:**
7	update Actor-Critic network of all agents
8	**end if**
9	**end for**

**Algorithm A4: Dual-APU-Ctrl:** Sharing Policy Algorithm	
1	Initialize an online Actor-Critic network
2	Initialize a target Actor-Critic network
3	**for** *episode* ← 0 **to** EPISODE
4	**for** *button* ← 1 **to** $N_{player}$
5	Play a board of Texas hold ’em as **Algorithm A1**, where all players act via sharing network
6	**end for**
7	**if** *episode* **mod** TRAINING_PERIOD **==0**:
8	update sharing Actor-Critic network
9	**end if**
10	**end for**
